# Primate Swallowing Is Powered by Both Rotation and Contraction of Suprahyoid Muscles

**DOI:** 10.1002/ajpa.70195

**Published:** 2026-01-10

**Authors:** Courtney P. Orsbon, Nicholas J. Gidmark, Callum F. Ross

**Affiliations:** ^1^ Department of Radiology University of Washington Medicine Seattle Washington USA; ^2^ Department of Organismal Biology & Anatomy The University of Chicago Chicago Illinois USA; ^3^ Biology Department Knox College Galesburg Illinois USA

**Keywords:** deglutition, diceCT, EMG, feeding, macaque, mammals, tongue base retraction, XROMM

## Abstract

**Objectives:**

Swallowing biomechanics in primates and other mammals is poorly understood, and the effect of hyoid descent on swallowing biomechanics lacks experimental interrogation. In macaques, which share similar swallowing kinematics with humans, the base of the tongue and the food bolus are hypothesized to be driven into the oropharynx by a hydraulic mechanism, at the core of which is elevation and protraction of the hyoid. Here, the musculoskeletal mechanisms driving these hyoid movements are experimentally evaluated in macaque primates.

**Materials and Methods:**

We integrate XROMM‐based measures of mandibular, cranial, and hyolingual kinematics with electromyography of suprahyoid and lingual muscles to evaluate the underlying kinetics of swallowing.

**Results:**

All suprahyoid muscles rotate during swallowing and tongue base retraction. Hyoid elevation and protraction are powered by concentric activation and rotation of posterior mylohyoid and both digastric muscle bellies, followed by concentric activation of geniohyoid muscle. Genioglossus is predominantly active early in swallowing.

**Discussion:**

Morphology, function, and coordination of suprahyoid and lingual muscles are especially important determinants of swallowing performance in macaques, and probably humans. Here, we characterize suprahyoid muscle biomechanics within a dynamic framework of architectural gear ratios and pulley systems that optimize hyoid elevation velocity to quickly “prime the pump” and subsequent hyoid protraction power and/or force to drive the hydraulic mechanism of tongue base retraction. Because of muscle rotation, primate hyolingual muscle function is particularly dependent on hyoid posture and muscle geometry, which may have important implications for the evolution of swallowing biomechanics in human evolution.

## 1 Introduction

Swallowing is a crucial function—hydration and nourishment must be safely directed from the oral cavity, bypass the larynx and lower respiratory tract, and ultimately enter the esophagus. Previous work has demonstrated how humans and other mammals achieve this feat through finely tuned sensorimotor integration in a manner not merely reflexive but rather modulated both over the course of ontogeny and in response to variable bolus properties in daily life (Doty and Bosma 1956, Dodds et al. 1988; Chi‐Fishman and Sonies 2002; Kajii et al. 2002; German et al. 2009; Steele et al. 2011; Humbert et al. 2012; Steele et al. 2012, Humbert et al. 2013, Selley et al. 1989; Smith et al. 1989; McFarland and Lund 1995; Crompton et al. 1997; German et al. 1998; Klahn and Perlman 1999; Martin‐Harris et al. 2003; Gewolb and Vice 2006; K. Matsuo et al. 2008; Matsuo and Palmer 2015; Ballester et al. 2018). Hence, safe swallowing is arguably a learned behavior—one is not born knowing how to wean from milk and safely swallow foods ordinarily consumed by adults, let alone pills.

**FIGURE 1:**
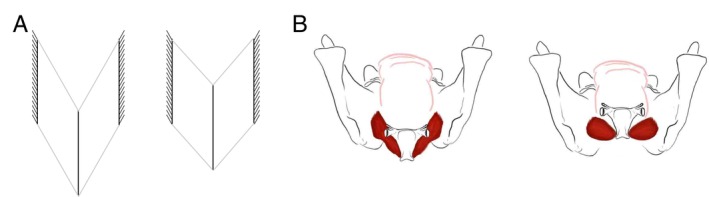
Diagram showing muscle architecture dynamics analogy. (A) Engineering model of the effects of how, in the setting of rigid and fixed lateral attachment points, rotation can facilitate linear displacement of a whole bipennate muscle through a combination of shortening and rotation. (B) Mylohyoid modeled as a bipennate muscle with fixed‐width origins, facilitating hyoid elevation through rotation. Necessary muscle fiber bulging could occur out of plane (anteroposteriorly) as well as superoinferiorly given that rigid mandibular attachment points prevent lateral bulge.

Learning to control the many muscles involved in swallowing over the course of weaning is complicated by how these structures change over the course of human ontogeny. Human infants are born with similar craniofacial and oropharyngeal anatomy compared to other nonhuman primates (Negus 1949; Crelin 1969; Falk 1973; Lieberman et al. 2001), but these structures grow in ways that have been hypothesized to make them maladapted to swallowing as a consequence of selection for traits that are adapted to speech (Negus 1949; Dantas et al. 1990; Lieberman et al. 1992; Lieberman et al. 2001). Specifically, the tongue, hyoid, and larynx (i.e., which originally functioned as a valve for airway protection during swallowing, Negus 1949) descend away from the hard palate and further into the neck (Lieberman et al. 2001) (Figure 1). This configuration separates the epiglottis and soft palate, which has been argued to be beneficial for easily producing unambiguous vowels in speech but dangerous for the airway because it allows greater mixing of air and food in the valleculae (Negus 1949; Laitman, Crelin, Conlogue, et al. 1977; Stevens 1989; Lieberman et al. 1992; Lieberman 2012).

However, other studies cast doubt on both of these claims, including studies of the physical capacity for vowel production in nonhuman primates (Fitch et al. 2016; Boë et al. 2017), the routine accumulation of food in the vallecular pouch prior to swallowing in humans (Palmer et al. 1992; Palmer 1998; Hiiemae and Palmer 1999; Matsuo and Palmer 2015), and the relatively rare incidence of choking‐related deaths in epidemiological studies (Heimlich 1974; Mittleman and Wetli 1982; Vilke et al. 2004; Soroudi et al. 2007; Inamasu et al. 2010; Sakai et al. 2014). As such, the relationship between the evolution of the human craniofacial traits and swallowing performance deserves reconsideration in a conceptual framework that integrates advances in biomechanics, experimentation, and mathematical modeling.

Compared to other behaviors such as chewing and locomotion, we know relatively little about the basic biomechanics of swallowing in humans and other primates. This knowledge gap in part reflects practical challenges to visualizing and measuring tongue, hyoid, and jaw kinematics. While limb kinematics can be observed using light cameras, the skin obscures the inner workings of the tongue and throat. Therefore, highly specialized—and not widely available—imaging equipment is necessary to visualize the swallowing structures with sufficient spatiotemporal resolution to effectively test hypotheses about swallowing biomechanics. Moreover, some of these approaches are sufficiently invasive or involve large doses of radiation such that healthy human subject research would be unethical. Second, limb and jaw biomechanics rest on a theoretical framework of lever mechanics, which allows biomechanical outputs such as force and velocity to be estimated from morphology (Smith and Savage 1956; Smith and Savage 1959). However, as will be discussed below, most of the structures involved in swallowing have no such levers, and the relationship between morphology and performance is less mathematically straightforward.

Given the difficulty of studying human swallowing biomechanics in vivo, a model organism with morphology and swallowing physiology similar to those of humans can establish a validated biomechanical framework for testing more general hypotheses about craniofacial and hyolingual form: function relationships, which would have broad utility in the fields of evolutionary biology and anthropology. For example, although masticatory adaptations have been studied extensively in primates and other mammals, whether swallowing performance has exerted similar selective pressure on hyolingual form and function is relatively unexplored. A better understanding of how craniofacial and hyolingual shape are related to swallowing may also help resolve long‐standing uncertainty about some relationships between primate craniofacial form and function (DuBrul and Sicher 1954; Daegling 1993; Dobson and Trinkaus 2002; Groening et al. 2011; Daegling 2012; Ross and Iriarte‐Diaz 2014).

The rhesus macaque ( *Macaca mulatta* ) is arguably a suitable model for human feeding in general (Hylander 1988; Haravu et al. 2022), and for swallowing in particular (Ross et al. 2024). Despite slight morphological differences in the swallowing structures, macaque swallowing physiology is similar to humans' (Franks et al. 1984; Franks et al. 1985; Palmer et al. 1992; Hiiemae et al. 1995; Matsuo and Palmer 2010; Nakamura et al. 2017; Hylander 1988; Haravu et al. 2022; Ross et al. 2024). Although hominoids (e.g., chimpanzees, bonobos, gorillas, gibbons) would arguably be better models because their laryngeal anatomy is more akin to humans than macaques (Nishimura 2003), the Great Ape Protection and Cost Savings Act of 2012 prohibits the use of great apes for this line of research. Moreover, rhesus macaques are a well‐established model system for human neurobiology, including orofacial motor control (Huang et al. 1989; Murray et al. 1991; Lin et al. 1994; Martin et al. 1997; Martin et al. 1999; Yao et al. 2002; Sessle et al. 2005; B. J. Sessle et al. 2007; Arce‐McShane et al. 2014; Arce‐McShane et al. 2016; Laurence‐Chasen et al. 2023). Overall, the use of rhesus macaques as a model for human swallowing biomechanics is a reasonable compromise among physiological similarity, phylogenetic relatedness, practical availability, and experimental rigor (Ross et al. 2024).

The research reported here is part of a project that uses the rhesus macaque model system to assess the biomechanical basis of the primate mechanism of tongue base retraction during swallowing and how craniofacial changes observed over human evolution and development affect swallowing performance. Previously, we developed a workflow to compare in vivo hyolingual muscle velocity and activity against craniofacial, hyoid, and tongue kinematics (Orsbon et al. 2018). This high accuracy and precision in vivo workflow integrates existing data acquisition workflows to simultaneously measure 3D mandibular and hyolingual kinematics with individual hyolingual muscle kinematics and activity. Specifically, the method integrates X‐ray Reconstruction of Moving Morphology (XROMM, Brainerd et al. 2010) with diffusible iodine‐based contrast enhanced computed tomography (diceCT; Mestcher 2009; Gignac et al. 2016) and electromyography (EMG; Loeb and Gans 1986).

The anatomy and physiology of the macaque feeding system and its relevance for human chewing and swallowing has been described elsewhere (Ross et al. 2024), hence only a brief review is provided here. The macaque mandible resembles that of humans in having a fused symphysis necessitating significant mediolateral jaw movement during chewing. Macaque and human hard palates lack strong transverse rugae, with implications for mechanisms of bolus transport during feeding discussed below.

Macaques and humans also share a fleshy spatulate tongue with a core of imbricated intrinsic vertical and transverse tongue muscles, covered superiorly by a layer of superior longitudinal muscles. The genioglossus muscles fan out into the tongue from the lingual surface of the symphysis, interweaving with the intrinsics; inferior longitudinal muscles lie on either side of this genioglossal stem, and extrinsic muscles merge into the lateral sides of the tongue.

This fiber arrangement appears to be conserved among studied anthropoid primates, although human tongues have a more rounded sagittal shape because of hyoid descent (Sokoloff et al. 2007; Takemoto 2001; Takemoto 2008). The intrinsic muscles are hypothesized to function as a muscular hydrostat in which fiber shortening produces movement by capitalizing on the incompressibility of the water within the muscle fibers—decreases in fiber length must be accommodated by bulging, which increases fiber width and depth (Kier and Smith 1985; Smith and Kier 1989). For example, tongue protrusion is hypothesized to result from a combination of transverse and vertical fiber shortening, which decreases tongue diameter and increases tongue length (Napadow et al. 1999).

Another set of muscles, the extrinsic muscles, originates from the cranium, mandible, hyoid, and soft palate. Styloglossus originates from the stylomandibular ligament in macaques and the styloid process in humans and inserts into the posterolateral tongue (Hartman and Straus 1933). Genioglossus originates from the mandibular symphysis and fans out in the midsagittal plane to insert into the tongue midline, interdigitating with the transverse and vertical fibers in the core of the tongue. Hyoglossus originates from the hyoid body and greater horns and inserts into the posterolateral tongue deep to styloglossus. Palatoglossus originates in the lateral part of the soft palate and inserts into the posterolateral tongue, merging with styloglossus. These extrinsic muscles have been argued to produce tongue movement by moving the entire tongue as well as by deforming the tongue locally. For example, genioglossus shortening is thought to produce a midline furrow in the tongue (Abd‐El‐Malek 1955; Stone and Lundberg 1996).

The primate hyoid is composed of several parts—a body, or basihyoid, a pair of lesser horns, and a pair of greater horns. Unlike many other mammals, and most lemuroids, the hyoid of haplorhine primates is not normally connected to the cranium by a chain of ossicles, but rather “floats,” suspended from the cranium, mandible, thyroid cartilage, pharynx, sternum, and scapula by a muscular sling (Hartman and Straus 1933; Sprague 1944; Li et al. 2022). Muscles originating from the cranium and mandible are broadly categorized as suprahyoid muscles, while muscles originating from the larynx and postcranial skeleton are termed infrahyoid muscles.

As discussed below, suprahyoid muscles produce hyoid movements during swallowing. Geniohyoid originates from the mandibular symphysis and inserts on the basihyoid. Mylohyoid originates on the lingual surface of the mandibular corpus along the mylohyoid line, and its posterior fascicles insert into the basihyoid whereas its anterior fascicles insert into a midline raphe. Stylohyoid originates from the styloid area in macaques and the styloid process in humans and inserts into the inferior pole of the basihyoid in macaques and at the junction between the basihyoid and greater horn in humans (Hartman and Straus 1933). Although neither belly of the digastric directly inserts into the hyoid, the digastric tendon is loosely attached to the hyoid by a membrane in macaques and a more robust fascial sling in humans and is therefore frequently considered a suprahyoid muscle (Hilloowala 1975). The posterior belly originates from the mastoid process of the basicranium in macaques and the mastoid notch in humans. In macaques, the anterior belly nearly completely obscures the anterior mylohyoid as it originates from the anterior half of the mandibular inferior border. However, in humans this belly is relatively small and inserts inferior to a more limited area at the mandibular symphysis in the digastric fossa (Hartman and Straus 1933).

During feeding in primates (as in most mammals), the tongue transports food from the ingestion point to the post‐canine dentition for chewing (stage I transport) (Franks et al. 1984; Franks et al. 1985; Hiiemae and Crompton 1985; German et al. 1989). Once the food bolus is on the post‐canine teeth, the tongue participates in mastication by working with the buccinator muscles of the cheek to keep food on the tooth row. Meanwhile, the highly sensitive lingual epithelium gathers sensory information to determine whether a bolus is ready to be swallowed (Hiiemae and Crompton 1985; Trulsson and Essick 1997; Mu and Sanders 2000; Hiiemae and Palmer 2003). The tongue transports the appropriately processed food bolus posteriorly into the valleculae (stage II transport), bilateral pouches between the posterior surface of the tongue and the epiglottis. After one or several of such stage II transport cycles, a swallow commences, and the tongue base retracts against the posterior wall of the pharynx, moving the bolus from the valleculae, through the pharynx, past a closed laryngeal inlet, and into the esophagus. Once complete, the tongue relaxes, the hyoid descends, and the larynx reopens as breathing resumes.

Movements of the tongue and hyoid are linked with those of the jaw as part of a “chain” in which the movements of one structure affect the movements of others (Hiiemae and Crompton 1985; Hiiemae and Palmer 2003), necessitating a basic understanding of jaw movements. During chewing and swallowing, mandibular movements increase gape through a combination of changes in pitch (i.e., rotation) and anterior translation at the temporomandibular joint (TMJ; Wall 1999; Keefe et al. 2008; Terhune et al. 2015; Ross et al. 2012; Menegaz et al. 2015; Iriarte‐Diaz et al. 2017). The cyclic movements of the mandible, the gape cycle—defined as the period from maximum gape to maximum gape—is often partitioned into four phases based on the velocity of the mandible: fast close (FC), slow close (SC), slow open (SO), and fast open (FO) (Hiiemae et al. 1995; Reed and Ross 2010; Iriarte‐Díaz et al. 2011; Ross et al. 2012; Nakamura et al. 2017). Humans and macaque monkeys also feature an intercuspal phase (IP), which overlaps with SC and SO, during which the molar cusps intersect and the mandible may cease to change in pitch (Palmer et al. 1992; Hiiemae et al. 1995; Orsbon et al. 2018).

In most studied nonprimate mammals, the bolus moves posteriorly as the hyolingual apparatus retracts during FO and FC, and ultimately masticated food accumulates in the valleculae (Franks et al. 1985; Hiiemae and Crompton 1985). Ridges on the hard palate called palatal rugae prevent anterior movement of the bolus as the tongue protracts during SO. During the swallow, the tongue base retracts during SO to transfer the bolus to the posterior oropharynx and laryngopharynx, after which the pharyngeal constrictors propel the bolus into the esophagus (Hiiemae and Crompton 1985).

In contrast, humans and macaques have diminutive palatal rugae that cannot resist anterior movement of the bolus as the tongue protracts during SO (Franks et al. 1984). Consequently, they transfer the bolus to the oropharynx using a different mechanism: the anterior tongue contacts the hard palate as the hyolingual apparatus protracts during IP and early SO; then, the middle tongue maximally elevates during SO; finally, the hyoid and posterior tongue are maximally elevated shortly after the SO‐FO transition (Nakamura et al. 2017). These movements create a wave of palatal contact traveling posteriorly, and the tongue “squeezes back” the bolus toward the valleculae (Franks et al. 1984; Palmer et al. 1992). As a result, stage II transport occurs during SO in catarrhine primates instead of FO and FC as it does in nonprimate mammals (Hiiemae and Crompton 1985).

Because humans and macaques store relatively small amounts of masticated food in their relatively small valleculae before the swallow as compared to other mammals, much of the masticated bolus is still in the oral cavity prior to swallowing (Franks et al. 1984; Palmer et al. 1992). When ready to swallow, the tongue performs a similar squeeze‐back mechanism that features greater hyoid excursion than a stage II transport, as well as tongue base retraction (Franks et al. 1984; Palmer et al. 1992). Unlike nonprimate mammals, humans and macaques use the squeeze‐back mechanism to swallow both food and liquids, whereas nonprimate mammals only use the squeeze‐back mechanism to transport liquids from the oral cavity to the valleculae (Hiiemae et al. 1981; Franks et al. 1984; Franks et al. 1985; Hiiemae and Crompton 1985; Palmer et al. 1992).

The squeeze‐back mechanism of primate tongue base retraction during swallowing has two important functions related to airway protection, both during and after the swallow. First, hyoid elevation and protraction pull the larynx superiorly and anteriorly to avoid contact with the oncoming bolus as tongue base retraction propels the bolus through the oropharynx and flips the epiglottis over the airway (McConnel 1988; Gassert and Pearson Jr. 2016). These movements function to prevent the bolus from entering the airway *during* the swallow. Second, as tongue base retraction propels the bolus toward the esophagus and clears the throat, or oropharynx, of any lingering residues (Dejaeger et al. 1997), hyoid protraction opens a sphincter guarding the entrance to the esophagus (Cook et al. 1989). This opening corresponds with increased pressures at the tongue base and laryngopharynx—likely facilitated by pharyngeal constrictor activity onset—which generates a pressure gradient that facilitates bolus transfer to the lower‐pressure esophagus and prevents bolus stasis in the laryngopharynx (McConnel 1988; Dejaeger et al. 1997). These movements function to prevent the bolus or its remnants from entering the airway once breathing resumes *after* the swallow.

Previous morphological and physiological research suggested that tongue base retraction is caused by shortening of the styloglossus, hyoglossus, and transverse intrinsic tongue muscles (Napadow et al. 1999; Gassert and Pearson Jr. 2016). Similarly, hyoid excursion correlates with geniohyoid, mylohyoid, and digastric morphology and activity (Palmer et al. 1992; Pearson Jr. et al. 2011; Inokuchi et al. 2014; Inokuchi et al. 2016). However, determining muscle function requires more than knowing the orientation of muscles and their activity patterns because muscle velocity (as a vector of both direction and magnitude) and activity must be measured simultaneously to determine whether the muscle shortens, lengthens, or is isometric during a given behavior, and whether it actively produces force in a given direction at that time (German et al. 2011). Moreover, changes in hyoid posture over smaller time scales, such as during a single feeding session, or over longer time scales, such as over human ontogeny or evolution, could affect these muscles' functions (German et al. 2011; Li et al. 2025).

When we tested hypotheses regarding the mechanism of tongue base retraction (Orsbon et al. 2020), we found that neither intrinsic nor extrinsic lingual muscle shortening alone or in combination could account for the extent of tongue base retraction or observations of regional volume changes within the tongue, which had been observed by other groups (Liu et al. 2008; Liu et al. 2009). We proposed that regional lingual volume displacement by suprahyoid muscles may produce tongue base retraction through a hydraulic linkage. In such a linkage, hyoid excursion and mouth floor elevation decrease the volume available for the tongue within the oral cavity. Consequently, some tongue volume must be displaced posteriorly because it cannot go superiorly due to the hard palate, anterolaterally because of the tooth row locked in the intercuspal phase, or inferiorly as the mouth floor both raises and stiffens because of mylohyoid and anterior digastric active shortening. In such a scenario, the hyoid effectively squeezes back the tongue, analogous to how the tongue squeezes back the bolus during swallowing.

The present study sought to better understand exactly how the hyolingual muscles function to elevate and protract the hyoid. If tongue base retraction is due to suprahyoid muscles producing hyoid excursion and mouth floor elevation as suggested above, then these muscles must be active during tongue base retraction. Hyoid elevation and protraction during swallowing are frequently assumed to result primarily from suprahyoid muscle active shortening based on human kinematic, morphologic, and EMG analysis (Pearson Jr. et al. 2011; Pearson Jr. et al. 2012; Pearson Jr. et al. 2013; Inokuchi et al. 2014; Inokuchi et al. 2016). Although hyolingual muscles are hypothesized to also behave isometrically and eccentrically (A. Thexton 1984), the potential functions of such activity are unknown. Moreover, the suspended geometric arrangement of hyolingual muscles unconstrained by joints allows hyoid movement to occur not only by muscle shortening but also muscle rotation that could, based on observations of pennate musculature, enhance hyoid displacement and velocity at the expense of muscle force production (Azizi et al. 2008). As with isometric and eccentric activity, the functional implications of muscle rotation are unclear.

A role for muscle rotation in hyoid movement is suggested by morphological similarities between the hyoid apparatus—a bone suspended by multiple muscles and unconstrained by joints—and pennate muscles (Figure 1), in which fiber rotation is an important component of skeletal muscle function in both locomotor and feeding systems (Brainerd and Azizi 2005; Azizi et al. 2008; Randhawa et al. 2013; Azizi and Roberts 2014; Holt et al. 2016; Laird et al. 2020; Laird et al. 2023). This research emphasizes that rotation of fascicles within pennate muscles enhances translational velocity of muscle end points at the expense of muscle force production. Such force‐velocity trade‐offs have been quantified with the architectural gear ratio (AGR) of whole muscle velocity to fascicle velocity. High AGRs are associated with greater degrees of fiber rotation for a given amount of whole muscle shortening than low AGRs, resulting in faster whole muscle shortening relative to fascicle shortening. The associated increases in pennation angle—the angle between muscle fascicles and the line of action, or force‐generating axis of the muscle—decrease the proportion of fascicle force along the muscle's line of action. As such, high AGRs are associated with lower muscle forces and greater muscle shortening velocities (Azizi et al. 2008). In contrast, low AGRs are associated with less muscle fiber rotation during shortening and result in smaller increases in pennation angles, lower muscle shortening distances and velocities, and higher force production.

Overall, the muscle gearing literature underscores the value of integrated morphologic and physiologic approaches to understanding muscle function from a dynamic perspective rather than inferring function from static morphology alone. Here, we apply the concept of the AGR from its description of muscle architecture dynamics within muscles to the level of whole muscle bellies of the floating—that is, joint‐free and less constrained—hyolingual apparatus. We use AGRs to quantify shortening and rotation of whole suprahyoid muscles and to estimate their contributions to hyoid kinematics during swallowing. We then use these AGRs to test the hypothesis that the hyoid will move faster than the suprahyoid muscles shorten because of muscle rotation.

In summary, the research reported here tests the following hypotheses:

*Suprahyoid muscles are active during tongue base retraction*.

*Hyolingual muscles are only active during muscle shortening*.

*Hyoid protraction, elevation, and total displacement are due only to geniohyoid and mylohyoid muscle shortening and are not amplified by geniohyoid and mylohyoid muscle rotation*.

## 2 Materials and Methods

Our workflow (Orsbon et al. 2018; Orsbon et al. 2020) integrates X‐ray Reconstruction of Moving Morphology (XROMM) (Brainerd et al. 2010) and diffusible iodine‐based contrast enhanced computed tomography (diceCT) (Metscher 2009; Gignac et al. 2016) with intramuscular fine‐wire electromyography (EMG) (Loeb and Gans 1986). The XROMM workflow was used to quantify 3D rigid body kinematics of the cranium, mandible, and hyoid (Brainerd et al. 2010; Knorlein et al. 2016). High‐resolution DiceCT and dissection were used to identify muscle attachments and to confirm marker and EMG electrode placement. The EMG data were used to assess the presence and relative magnitude of muscle activation during swallowing. All procedures were approved by the University of Chicago IACUC.

### 2.1 Animal Subjects

Four adult rhesus macaques ( *Macaca mulatta* ) were used in this study. In accordance with the principle of animal subject reduction in animal research ethics, the monkeys had been used previously in neurophysiological experiments involving neural array implantation in orofacial motor and sensory cortex (Monkey H, female, age 8, 7.46 kg), limb areas of sensorimotor cortex (Monkey K, female, age 12, 7.55 kg), parietal and premotor cortex (Monkey J, male, age 16, 8.48 kg), and prefrontal cortex (Monkey C, male, age 9, 8.83 kg). The animals were housed in an AAALAC‐accredited animal facility attended to daily by veterinary and husbandry staff. The animals were trained to be transferred to and from their cages by pole‐and‐collar and to feed while restrained in a radiolucent, acrylic primate chair. On non‐training days, animals were fed monkey biscuits and given daily enrichment along with ad libitum access to water. On training days, the animals were food and water delayed until after data collection to facilitate data collection as needed. To control for bolus material properties, feeding trials of multiple fruits and vegetables were conducted to determine a commonly preferred food among the four animals, and the monkeys were all fed red grapes with skin cut to approximate 1 cc. Before proceeding with surgical instrumentation, the animals were screened for laryngeal penetration and/or aspiration under fluoroscopy while swallowing iodine and/or barium‐containing liquids (discussed below). Screening was negative for all animals.

On the day of surgery, the animals were sedated under general anesthesia, intubated, shaved, and prepared in a sterile fashion within a dedicated veterinary operating room. Dissection and bone drilling were performed to implant 1.0 mm tantalum markers in the cranium and mandible (Orsbon et al. 2018). The same method was used to implant markers in the hyoid body when sufficient bone stock was available in this thin bone. An intraoperative decision was required to assess whether a secondary method of suturing laser‐drilled tantalum beads to the inferior pole of the hyoid would be required (discussed in detail with sensitivity analysis in Orsbon et al. 2018). Angiocatheters, 16G, were used to implant markers in the lingual submucosal tissues. Methods and marker constellations are documented in Orsbon et al. 2018 and 2020. For EMG recordings, insulated, medical‐grade, multi‐stranded stainless‐steel fine‐wire electrodes (Cooner Wire, Chatsworth CA) were chronically implanted in muscles. After stripping 1–2 mm of insulation from the ends of the wires, the electrodes were implanted into the muscles using an 18‐ or 20‐gauge needle. Location was confirmed using back‐stimulation (single stimulation, train rate = 1 Hz, train duration = 300 ms, stim rate = 250 pulses per second, delay = 0.01 ms, duration = 0.2 ms, voltage = 5–10 V) (Grass S48 Stimulator, AstroNova Inc., West Warwick RI). The wires were secured to surrounding connective tissues with 4–0 Vicryl and tunneled to a 27‐pin connector (Omnetics Connector Corporation, Minneapolis MN) housed in a custom‐built, percutaneous, titanium housing rigidly fixed to the cranium. After confirming hemostasis, incisions were closed with interrupted deep dermal sutures and running subcuticular sutures to prevent the animal from picking at exposed sutures postoperatively. The animals received postoperative intramuscular buprenorphine for 2 days and cephalosporin antibiotics for 5–7 days. Two weeks elapsed prior to collecting data to allow healing and formation of scar tissue around the markers, fixing them in place relative to surrounding tissues. Comparisons of fluoroscopic observations using contrast‐enhanced liquids (240 mg/mL iohexol and/or 60% w/v barium sulfate, depending on animal preference) mixed in a 1:1 mixture of fruit juice after surgery revealed no evidence of aspiration or penetration after these procedures. Minimal, if any, residue in the pharynx was noted after these contrast‐enhanced swallows, which was to be expected given minimal residue seen in humans swallowing 60% w/v and no residue seen in humans swallowing 22% w/v barium sulfate (Steele et al. 2013). Animals were weighed multiple times per week and assessed by staff multiple times daily. No animals exhibited significant weight loss or were treated for pneumonia in the postoperative period.

### 2.2 Data Collection

Kinematic and EMG data were collected in the University of Chicago's XROMM Facility (https://xromm.uchicago.edu/). Biplanar videoradiographic data were collected at 200 Hz, at 90–100 kVp and 10–16 mA, and with a 2000–4000 microsecond shutter speed. Electromyographic signals were obtained from electrodes implanted unilaterally and in series along the long axis of the muscle bellies of the posterior mylohyoid (all monkeys), geniohyoid (all monkeys), genioglossus (all monkeys), anterior digastric (monkeys C, H, and J), styloglossus (monkey C), and posterior digastric (monkey J). Attempts were made at implanting electrodes in additional suprahyoid muscles in all animals, but the data from these electrodes were excluded if found to be outside of the muscle bellies postmortem (methods described in part below and in more detail in Orsbon et al. 2018), presumably due to extrusion over the course of healing. Signals from verified intramuscular electrodes were amplified (gain: 100–10,000), bandpass filtered (high‐pass: 1–10 Hz; low‐pass: 1–5 kHz) (AM Systems Model 1700), and recorded at 2–10 kHz using a video processing and recording unit (Xcitex ProCapture VPU) that synchronized the video and EMG data sources. At the conclusion of data collection, the animals were placed under deep anesthesia, the cardiac left ventricle was exposed and cannulated, and the animal was perfused under pressure with 0.5–1.0 L of saline plus heparin sodium (10 IU/mL) followed by 1.0–2.0 L 10% formalin solution, and finally sacrificed by exsanguination via a right atrial incision. To preserve a more neutral hyoid position to measure post‐mortem muscle length, connections between the sternohyoid and the sternum were maintained throughout fixation.

### 2.3 CT Data Collection and Processing

Methods are described in detail Orsbon et al. (2018) and are adapted from approaches described in Gignac et al. (2016). The head and neck specimens were immersed in 20 L of 10% formalin solution for at least 7 days to fix and decontaminate the tissue before scanning in the PaleoCT at the University of Chicago (General Electric Phoenix v|tome|x Microfocus CT (microCT) scanner) (http://luo‐lab.uchicago.edu/paleoCT.html). The specimens were then placed in a 5 L 20% weight/volume sucrose solution prewash to minimize tissue shrinkage during staining (Morhardt and Witmer 2016). Following the prewash, the specimens were placed in a 1.25% I_2_/2.50% KI (iodine‐containing) solution then scanned every 2–4 weeks to evaluate staining progress. After at least 2 weeks in 1.25% I_2_/2.50% KI solution, the specimens were transferred to a 2% I_2_/4% KI solution that was replenished every 2–4 weeks until staining of the center of the specimen was observed on the microCT scans. The specimens were then placed in a deionized water bath for 2–4 days to evenly distribute contrast throughout the specimen before the final microCT scan.

The final microCT data were segmented using Amira 5.5.0 (FEI Company, Hillsboro OR). Fascicles were defined as the high‐density material (muscle fibers and endomysium) surrounded by lower density material (perimysium). Low‐density tissues below a minimum gray‐scale value threshold were excluded from the segmented volume. No distinction could be made between fibers of styloglossus because voxel width approximated the reported diameter of macaque styloglossus fibers (37.0–64.5 μm vs. ca. 40–90 μm, respectively; Sokoloff et al. 2007). The entire stylohyoid, palatoglossus, and posterior digastric muscles were segmented. In those muscles with EMG electrodes (see above), the fascicles immediately adjacent to the electrodes' bare ends were segmented out to their bony attachments. One exception is the posterior digastric in monkey J. Given that the posterior digastric belly is a bipennate muscle, the entire muscle belly attachment site was used. In hyoglossus, genioglossus, styloglossus, and anterior digastric, the fascicles containing or most closely related to implanted lingual markers were segmented. Muscle attachment locations on bone models were identified from segmented scans and dissection. The tantalum markers in the cranium, mandible, and hyoid were segmented for registration with the unstained bone model dataset using the AffineRegistration function in Amira (Orsbon et al. 2018).

### 2.4 Kinematic Analysis

Bone models were segmented to create polygonal mesh models using Amira 5.5.0 hosted on a visualization node of the Research Computing Center at the University of Chicago. 3D rendered figures were based on a shell created using 3‐matic Research v10.0 (Materialise, Leuven), smoothed and with metal implant artifacts removed using Autodesk ReMake 2016 (Autodesk, San Rafael CA).

Jaw gape cycles were defined from maximum gape to maximum gape using mandibular pitch. Swallow cycles were identified by the passage of the bolus through the oropharynx, determined to be when the radiographic shadows of the bolus, tongue, soft palate, and pharynx merged, accompanied by marked elevation of the hyoid and thyroid (Nakamura et al. 2017). The onset of TBR was defined as the time at which the posterior superficial tongue marker was at its most anterior point before beginning to move posteriorly. The offset of TBR was defined as the time when the vallecular marker was at its most posterior point after TBR onset.

Prior work utilized cranial and mandibular coordinate systems established from the XYZ coordinates of the landmarks on the bones using custom‐written scripts in R (R Core Team 2017; Orsbon et al. 2018). In the cranial coordinate system, the transverse (XZ) plane is parallel to the occlusal surface of the maxillary tooth row (positive X = anterior, positive Y = superior, positive Z = right) and the origin is at the posterior nasal spine. In the mandibular coordinate system, the transverse plane is parallel to the occlusal surface of the mandibular molars, and the origin is halfway between the mandibular condyles (Ross et al. 2012; Ross and Iriarte‐Diaz 2014; Menegaz et al. 2015; Iriarte‐Diaz et al. 2017; Nakamura et al. 2017; Orsbon et al. 2018; Orsbon et al. 2020). Data presented here are in a mandibular coordinate system to isolate hyoid kinematics at the low gape angles observed during tongue base retraction.

The relationships of hyoid kinematics to suprahyoid muscle length and rotation during TBR were quantified using changes in extrinsic muscle length and orientation quantified as the difference in their values between TBR onset and offset. Muscle length (d) was measured as the Euclidean distance between muscle attachment points. Genioglossus length and orientation were measured from the tendinous attachment on the lingual surface of the symphyseal region of the mandible to a marker within a genioglossus fascicle. Hyoglossus length and orientation were measured from attachment points on the hyoid to a marker within a hyoglossus fascicle. The digastric lengths and orientations were measured from attachments on the mandible (anterior digastric muscle) and cranium (posterior digastric muscle) to a marker implanted in the digastric tendon. Styloglossus length and orientation were measured from a marker in the lateral tongue to an estimated location for its attachment to the stylomandibular ligament (Orsbon et al. 2018). Muscle lengths were normalized using post‐mortem distances with animals fixed in a neutral posture, with the neck slightly flexed, the midline of the face in the midsagittal plane, the mandible slightly depressed (ca. 5 mm of gape at the incisors), and the tongue slightly protruded.

Muscle orientation vectors were expressed using anteroposterior (X), superoinferior (Y), and mediolateral (Z) components in a mandibular coordinate system, with positive values oriented anteriorly, superiorly, and right. Muscle angles were calculated in the sagittal plane (XY) from the right lateral view for geniohyoid, mylohyoid, stylohyoid, hyoglossus, and both digastric muscles and in the coronal (ZY) plane from the posterior view for mylohyoid, with 0° indicating anterior in sagittal planes and right in coronal planes. Muscle linear velocity was calculated by taking the first derivative of the data using the splinefun function of the R stats package (R‐Development‐Core‐Team 2010).

The impacts of geniohyoid and mylohyoid muscle shortening and rotation on hyoid displacement and velocity were calculated using average and peak suprahyoid architectural gear ratios (AGRs). As with prior work (Brainerd and Azizi 2005; Azizi et al. 2008; Laird et al. 2020), our AGRs are ratios of hyoid velocity to muscle velocity. AGR was calculated as the ratio of sagittal plane hyoid displacement—measured by the Euclidean (XY) distance between hyoid position—to muscle shortening from hyoid movement onset to offset. These displacements were measured across the same time frame; therefore, average velocity measurements were not required because time cancels out of the equation. An additional AGR was measured because geniohyoid and mylohyoid did not show distinct periods of constant velocity. This second AGR (AGRp) compares peak hyoid and muscle shortening velocities measured at the time of peak muscle shortening velocity.

Muscle AGR was also applied slightly differently in this study because of two‐dimensional hyoid kinematics, as opposed to once dimensional systems employed by much of prior work. Moreover, the study assessed hyoid kinematics from the perspective of muscle shortening and rotation alone rather than muscle bulging, particularly given the lack of tight fascial muscle compartments in the suprahyoid neck. Average AGRs were calculated along X and Y axes (${\mathrm{AGR}}_{\underline{X}}$ and ${\mathrm{AGR}}_{\underline{Y}}$) as the ratios of hyoid displacement along the respective axis to muscle length changes across the period from hyoid movement onset to offset along these axes. Peak AGRs ($A{\mathrm{GR}}_{\mathrm{Xp}}$ and $A{\mathrm{GR}}_{\mathrm{Yp}}$) were also calculated as the ratio of hyoid velocity along the respective axis to muscle velocity at the time of peak muscle shortening velocity.

### 2.5 Electromyography

Electromyography (EMG) signals were processed with a 30 Hz high‐pass Butterworth filter using the *butter* and *filtfilt* functions of the signal R package (Signal developers 2013). Low‐pass cutoffs ranged from 1 to 3 kHz, depending on experiment‐specific sample rates (2–10 kHz). Filtered data were full‐wave rectified and integrated using a root‐means squared (RMS) algorithm integrated over 5 ms intervals, and the data down sampled to match the videoradiography frame rate of 200 Hz. Each channel's noise threshold was determined using a version of Thexton's runs test method (A. J. Thexton 1996), modified to use an average threshold of 30 runs tests for each channel, and a threshold from a temporal subset of each trial in some channels (Orsbon et al. 2018). To calculate average muscle activity and velocity across all swallow cycles, EMG data were smoothed with a 50 Hz low‐pass Butterworth filter in 5 ms intervals from 200 ms before to 200 ms after the onset of tongue retraction.

To evaluate the importance of muscle contraction for driving hyoid movement during swallowing, isometric, concentric, and eccentric muscle activity were defined as muscle activity at or above 5% of noise threshold when muscle velocity was, respectively, within, below, or above two standard deviations of precision study velocity of zero (Orsbon et al. 2018; Orsbon et al. 2020).

## 3 Results

DiceCT scans confirmed EMG electrode placement in the anterior digastric, genioglossus, geniohyoid, and mylohyoid muscles in all four animals. Electrodes originally placed in stylohyoid and hyoglossus muscles were found to have at least one wire outside of the muscle; therefore, these EMG channels were not included in analysis. Monkey C had confirmed electrodes in styloglossus, and Monkey J had confirmed electrodes in posterior digastric. As the pilot animal in this experiment, Monkey K did not have markers implanted in the digastric tendon; therefore, anterior digastric length and velocity data were not available for this animal. Values are reported as median or median ± standard deviation.

### 3.1 Suprahyoid and Lingual Muscles Are Active and Rotate Throughout Tongue Base Retraction

**FIGURE 2:**
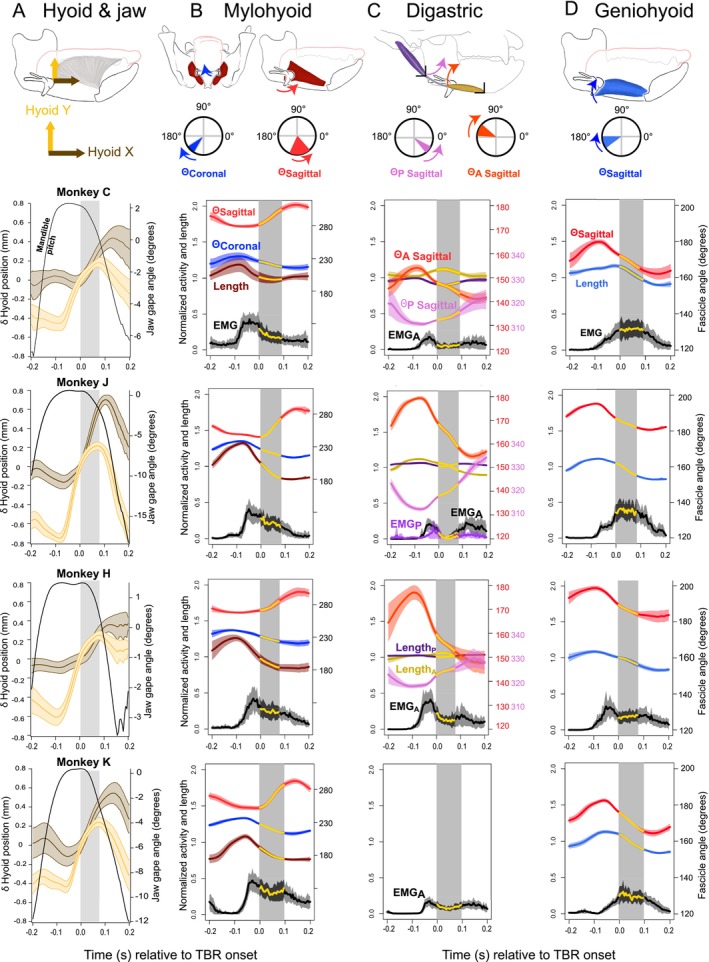
Hyoid kinematics and normalized activity, normalized length, and orientation of mylohyoid, digastric, and geniohyoid during swallow cycles in a cranial coordinate system. Data for muscles are in columns; data for individual animals are presented in rows (Monkeys C, J, H, K). (A) Hyoid. (B) Mylohyoid. (C) Digastrics. (D) Geniohyoid. No muscle kinematic data are available for the digastric of Monkey K. Top figures: hyoid and muscle location; for muscles, compass illustrates coordinate system and range of measured muscle fascicle orientation through the swallow. Curved arrow on compasses indicates direction of change in fascicle orientation vector during tongue base retraction (TBR). Bottom figures: (A) Changes in hyoid X and Y normalized to TBR onset. (B–D) Normalized EMG traces are shown in black. Normalized lengths (dimensionless proportion of *post mortem* length) are in color of muscle in top figure. Sagittal orientation angles are shown in red—more negative values indicate a more superoinferior orientation. In all graphs, dark traces are mean values; lighter shades are standard deviations. Vertical gray boxes indicate the mean duration of TBR and coincide with the yellow portions on the muscle length and EMG traces. Data are aligned to the onset of TBR (Time = 0.0).

**FIGURE 3:**
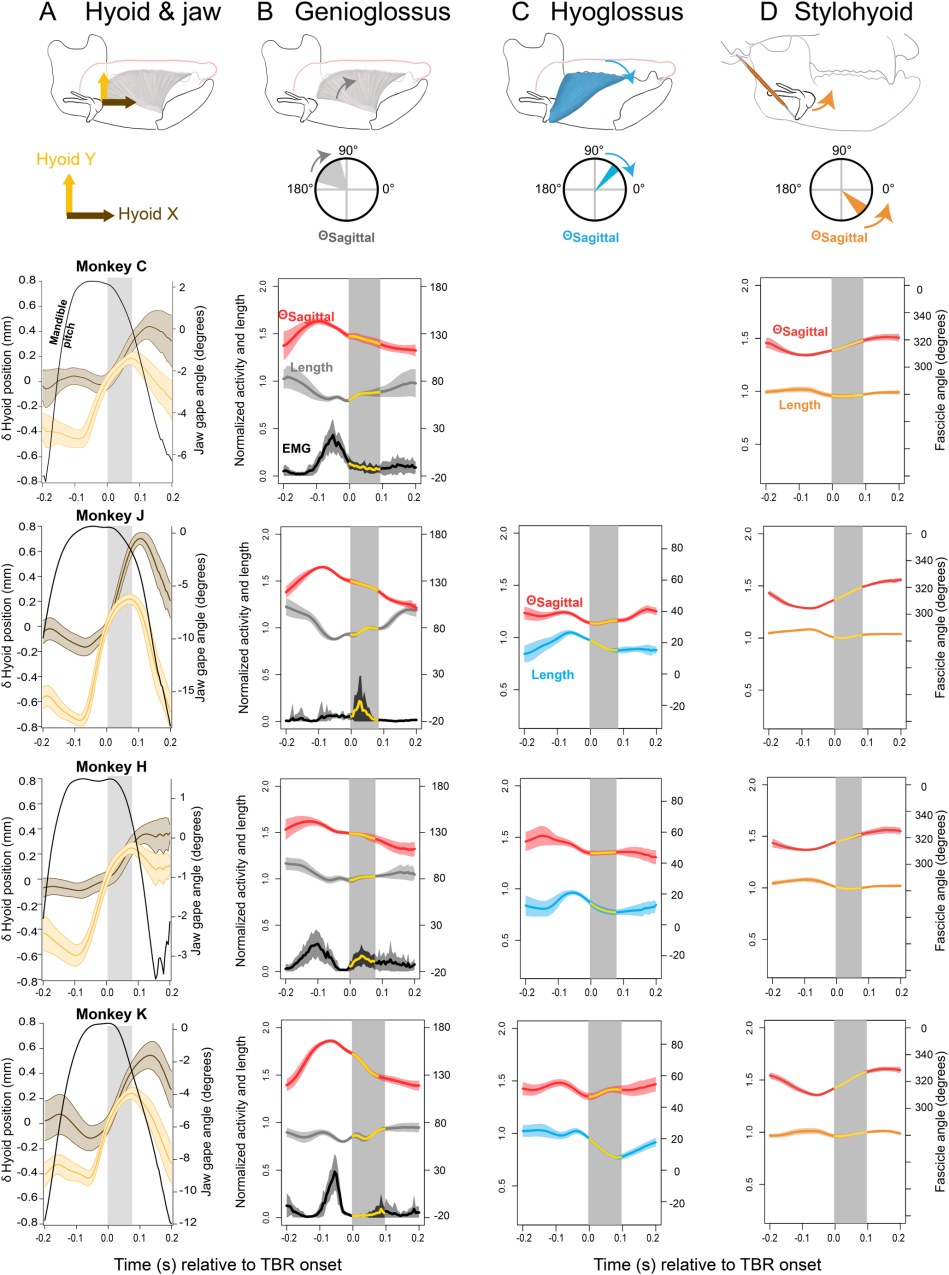
Hyoid kinematics and normalized activity, normalized length, and orientation of genioglossus, geniohyoid and hyoglossus muscles during swallow cycles in a cranial coordinate system. Data for hyoid and muscles are in columns. (A) Hyoid. (B) Genioglossus. (C) Hyoglossus. (D) Stylohyoid. Data for individual animals (C, J, H, K) are presented in rows. No hyoglossus data are available for Monkey C. Top figures: hyoid and muscle location; for muscles, compass illustrates coordinate system and range of measured muscle fascicle orientation through the swallow. Curved arrow on compasses indicates direction of change in fascicle orientation vector during tongue base retraction (TBR). Bottom figures: (A) Changes in hyoid X and Y normalized to TBR onset. (B–D) Normalized EMG traces are shown in black. Normalized lengths (dimensionless proportion of *post mortem* length) are in color of muscle in top figure. Sagittal orientation angles are shown in red—more negative values indicate a more superoinferior orientation. In all graphs, dark traces are mean values; lighter shades are standard deviations. Vertical gray boxes indicate the mean duration of TBR and coincide with the yellow portions on the muscle length and EMG traces. Data are aligned to the onset of TBR (Time = 0.0).

Activity, length, and orientation of geniohyoid, posterior mylohyoid, and digastric muscles during swallows are summarized in Figure 2, with the gray boxes in each graph indicating TBR. As shown in the left‐hand column, TBR was accompanied by upward and forward displacement of the hyoid, even as gape increased throughout TBR. Hyoid elevation began prior to TBR and then combined with hyoid protraction at or just before the onset of TBR (Figures 2A and 3A) (Orsbon et al. 2020). For all muscles below, references to rotation in sagittal planes are from the right lateral view, as shown in Figures 2 and 3.

Prior to swallowing, posterior mylohyoid ran posteroinferiorly at 260.54° in sagittal planes and inferomedially at 237.43° in coronal planes, from its origin on the alveolar prominences to its insertion on the hyoid. At the onset of swallowing and continuing throughout TBR, posterior mylohyoid actively shortened while its hyoid attachment swung counter‐clockwise 37.73° in sagittal planes, bringing the hyoid anterior to its origin, and clockwise −22.03° in coronal plane (Table 1) from posterior view, with the hyoid remaining inferior to its origin (Figure 2B).

**TABLE 1:** Suprahyoid muscle rotational kinematics.

Measurement	All monkeys	Monkey C	Monkey H	Monkey J	Monkey K
Mylohyoid					
Coronal angle start	237.43° (4.07°)	234.9° (5.89°)	240.05° (2.27°)	238.84° (1.06°)	236.27° (1.72°)
Coronal angle end	215.40° (4.41°)	214.79° (3.44°)	218.43° (4.80°)	213.11° (1.48°)	212.05° (2.95°)
Coronal rotation	−22.03°	−20.11°	−21.62°	−25.73°	−24.22°
Sagittal angle start	260.54° (13.53°)	278.17° (3.32°)	267.65° (1.82°)	245.07° (0.97°)	251.04° (3.26°)
Sagittal angle end	298.27° (10.56°)	313.50° (3.85°)	302.87° (7.98°)	288.69° (3.68°)	294.63° (4.40°)
Sagittal rotation	37.73°	35.33°	35.22°	43.62°	43.59°
Anterior digastric					
Sagittal angle start	169.38° (11.05°)	154.19° (1.82°)	174.72° (2.34°)	179.54° (0.89°)	
Sagittal angle end	148.29° (6.06°)	140.95° (2.10°)	147.24° (3.56°)	154.50° (1.08°)	
Sagittal angle rotation	−21.09°	−13.24°	−27.48°	−25.04°	
Posterior digastric					
Sagittal angle start	313.92° (3.68°)	310.54° (0.93°)	318.66° (0.93°)	312.47° (0.83°)	
Sagittal angle end	327.24° (6.07°)	319.97° (3.39°)	331.22° (3.91°)	331.06° (1.90°)	
Sagittal angle rotation	13.32°	9.43°	12.56°	18.59°	
Stylohyoid					
Sagittal angle start	308.37° (2.72°)	308.42° (1.56°)	310.83° (1.00°)	304.46° (0.76°)	309.70° (1.20°)
Sagittal angle end	325.52° (3.34°)	322.54° (2.14°)	326.50° (2.83°)	324.49° (1.28°)	329.98° (1.73°)
Sagittal angle rotation	17.15°	14.12°	15.67°	20.03°	20.28°
Genioglossus					
Sagittal angle end	147.62° (11.07°)	141.95° (2.89°)	137.76° (4.41°)	144.35° (1.38°)	165.95° (1.58°)
Sagittal angle start	118.96° (6.89°)	115.21° (3.05°)	115.74° (5.53°)	116.09° (1.86°)	129.16° (1.83°)
Sagittal angle rotation	−28.66°	−26.74°	−22.02°	−28.26°	−36.79°
Geniohyoid					
Sagittal angle start	189.28° (8.18°)	180.25° (1.65°)	199.04° (1.25°)	195.39° (0.58°)	182.51° (1.08°)
Sagittal angle end	172.2° (9.53°)	161.02° (2.70°)	181.38° (2.88°)	180.49° (0.69°)	164.10° (1.71°)
Sagittal rotation	−17.08	−19.23°	−17.03°	−14.90°	−18.41°
Hyoglossus					
Sagittal angle start	40.34° (6.55°)		44.39° (3.54°)	31.83° (0.70°)	45.35° (1.94°)
Sagittal angle start	50.60° (7.51°)		55.06° (4.34°)	40.65° (2.13°)	55.54° (2.21°)
Sagittal rotation	10.26°		10.67°	8.82°	10.19°

*Note:* Angles measured in degrees, with coronal angles measured from the posterior view and sagittal angles measured from the right lateral view, as demonstrated in Figures 2 and 3. Angles are measured from the start to the end of swallowing, as measured by the onset and offset of hyoid excursion in the X and Y axis.

As in humans, the anterior and posterior digastric muscles of macaques are joined by a short tendon. Prior to swallowing, the anterior digastric muscle ran posteriorly from its origin on the anterior hemimandible to its implanted tendinous marker near the hyoid body, nearly parallel with the anteroposterior (X) axis at 169.38° in sagittal planes. Meanwhile, the posterior digastric muscle ran anteroinferiorly from its origin on the basicranium to its implanted tendinous marker near the midportion of the greater horn of the hyoid at 313.92° in sagittal planes. Anterior digastric behavior was variable before TBR but consistently actively shortened by late TBR, whereas the posterior digastric muscle actively shortened before and then lengthened with minimal but suprathreshold activity during TBR. During swallowing and throughout TBR, the anterior digastric muscle rotated clockwise −21.09° and the posterior digastric muscle rotated counterclockwise 13.32°, forming an angle of 178.95° between the two muscles by the end of TBR. Stated qualitatively, mandibular depression and hyoid protraction and elevation were accompanied by reciprocal rotation of the tendinous attachments of the two digastric bellies, causing them to be nearly collinear by the end of TBR (Figure 2C).

Prior to swallowing, the geniohyoid muscle ran posteriorly and slightly inferiorly from its origin on the lingual mandibular symphysis to its insertion on the hyoid, 189.28° in sagittal planes. At the onset of swallowing and throughout TBR, the geniohyoid muscle predominantly actively shortened and rotated clockwise −17.08° in sagittal planes, with the highest concentric activity synchronous with TBR in 3 of 4 animals (Figure 3C, Table 1).

Similar to Figure 2, the activity, length, and orientation of genioglossus, hyoglossus, and stylohyoid during swallows are summarized in Figure 3, again with the gray boxes in each graph indicating TBR.

**FIGURE 4:**
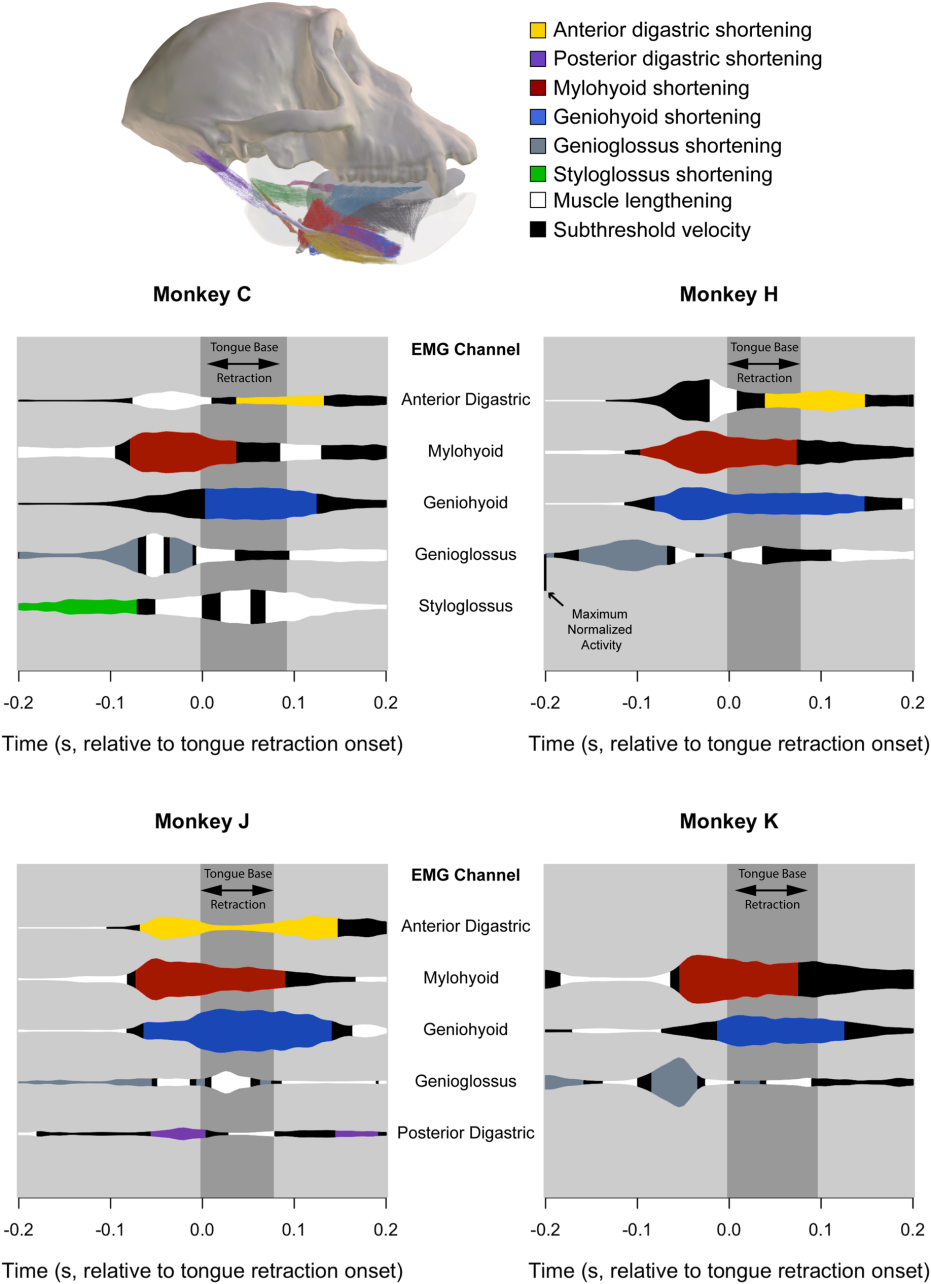
Isometric, concentric and eccentric hyolingual muscle activity during swallow cycles. Y‐axis is normalized EMG activity; X‐axis is time (s) relative to onset of tongue base retraction (TBR) (Time = 0.0); vertical dark gray bar indicates the duration of TBR. Black, isometric muscle activity (activity while muscle velocity below threshold); white, eccentric activity (activity while lengthening); colors, concentric activity (activity while shortening). Data are not available for some animals because they either lacked markers (Monkey K digastrics) or because correct EMG electrode placement was not verified. Modified from Orsbon et al. (2020).

Prior to swallowing, the genioglossus muscle ran posterosuperiorly 147.62° in sagittal planes from its origin at the mandibular symphysis to its insertion in the midline tongue. This angle varied across individuals because of inter‐individual variation in the position of intra‐lingual markers in genioglossus. At the onset of swallowing and throughout TBR, the genioglossus muscle rotated clockwise −28.66° in sagittal planes (Figure 3B). Although there were variable patterns of activity and length change among the four animals, there was relatively consistent concentric activity primarily observed prior to TBR near the onset of hyoid elevation (Figure 4).

Prior to swallowing, the hyoglossus muscle ran anterosuperior from its origin on the hyoid to its insertion in the lateral tongue, 40.34° in sagittal planes. At the onset of swallowing, the hyoglossus muscle shortened and rotated clockwise and then minimally counterclockwise during TBR, resulting in an overall clockwise rotation of −10.26° over the course of the swallow (Figure 3D, Table 1).

Prior to swallowing, the stylohyoid muscle ran posteroinferiorly 308.37° in sagittal planes from its origin on the basicranium to its insertion on the hyoid, similar to the posterior digastric muscle. During early swallowing as the hyoid elevated, the stylohyoid muscle shortened and rotated counterclockwise. Again, no reliable EMG data were obtained for this muscle. During TBR as the hyoid began to protract, the stylohyoid tended to lengthen minimally as counterclockwise rotation continued. Throughout swallowing and TBR, stylohyoid rotated counterclockwise 17.15° in sagittal planes (Figure 2D, Table 1).

### 3.2 Hyoid Velocity and Displacement Are Amplified by Mylohyoid Muscle Rotation

Table 2 includes architectural gear ratios (AGR) in four rows each for mylohyoid and geniohyoid: AGR, AGR at the time of peak muscle shortening velocity (AGRp), AGRs calculated along X or Y axis (${\mathrm{AGR}}_{\underline{X}}\;\text{or}\;{\mathrm{AGR}}_{\underline{Y}}$, respectively), and AGRs along X or Y axes calculated at the time of peak muscle shortening velocity ($A{\mathrm{GR}}_{\mathrm{Xp}}$ or $A{\mathrm{GR}}_{\mathrm{Yp}}$, respectively).

**TABLE 2:** Suprahyoid muscle gearing.

Measurement	All monkeys	Monkey C	Monkey H	Monkey J	Monkey K
Mylohyoid					
AGR	2.35 (1.70)	3.37 (2.68)^a^	1.55 (0.11)	1.68 (0.05)	2.01 (0.13)
AGRp	1.26 (0.22)	1.30 (0.36)^a^	1.13 (0.12)	1.25 (0.05)	1.37 (0.10)
AGR_Y_	1.67 (0.90)	2.03 (1.37)^a^	1.30 (0.11)	1.25 (0.04)	1.42 (0.13)
AGR_Yp_	1.22 (0.20)	1.29 (0.31)^a^	1.11 (0.13)	1.21 (0.06)	1.31 (0.12)
Geniohyoid					
AGR	1.51 (0.21)	1.72 (0.27)	1.45 (0.15)	1.36 (0.03)	1.45 (0.12)
AGRp	1.17 (0.11)	1.21 (0.12)	1.12 (0.09)	1.08 (0.03)	1.23 (0.07)
AGR_X_	1.00 (0.14)	1.15 (0.07)	0.79 (0.05)	0.94 (0.01)	1.10 (0.03)
AGR_Xp_	1.04 (0.07)	1.11 (0.07)	0.95 (0.03)	1.01 (0.01)	1.09 (0.02)

*Note:* AGR was calculated for geniohyoid and mylohyoid, calculated as the ratios of hyoid sagittal plane displacement to muscle length changes from the hyoid onset to offset. Peak AGR (AGRp) was calculated at the time of peak muscle shortening velocity. Average AGRs were calculated along separate X and Y axes (${\mathrm{AGR}}_{\underline{X}}$ and ${\mathrm{AGR}}_{\underline{Y}}$) as the ratios of hyoid displacement to muscle length changes from hyoid movement onset to offset on each axis. Peak AGRs ($A{\mathrm{GR}}_{\mathrm{Xp}}$ and $A{\mathrm{GR}}_{\mathrm{Yp}}$) were calculated at the time of peak muscle shortening velocity.

^a^ Mylohyoid AGR and ${\mathrm{AGR}}_{\underline{Y}}$ in Monkey C was significantly different between swallows looking right versus left. Reporting as mean (SD) and comparing using the Wilcoxon Signed‐Rank Test, *ad hoc* analyses showed that mylohyoid AGR looking right was 2.14 (0.17) versus looking left 5.6 (2.7), *p* < 0.001. Mylohyoid AGRp looking right was 1.07 (0.12) versus looking left 1.47 (0.39), *p* < 0.001. Mylohyoid ${\mathrm{AGR}}_{\underline{Y}}$ looking right was 1.63 (0.21) versus looking left 3.43 (1.35), *p* < 0.001. Mylohyoid AGR_Yp_ looking right was 1.04 (0.12) versus looking left 1.43 (0.29). In contrast, no measurement for geniohyoid was significantly different between looking right and looking left, *p* > 0.20.

Mylohyoid and geniohyoid AGRs demonstrate that sagittal plane hyoid translation from swallowing onset to offset was greater than either muscle shortened (2.35 and 1.51, respectively). Gearing was less pronounced in mylohyoid and geniohyoid peak velocity (AGRp, 1.26 and 1.17, respectively). However, given the two‐dimensional nature of hyoid kinematics and the more linear arrangement of nearly perpendicularly oriented geniohyoid and mylohyoid, these AGRs could be inflated simply because a hypotenuse will be greater than either side of a triangle. Because the line of action for mylohyoid is centered on the Y‐axis and the line of action for geniohyoid is centered on the X‐axis, separate AGRs along these axes to assess effects of rotation on hyoid elevation and protraction separately were calculated.

Hyoid elevation, and to a lesser degree, hyoid peak elevation velocity, are amplified by posterior mylohyoid muscle rotation. The median posterior mylohyoid ${\mathrm{AGR}}_{\underline{Y}}$ was 1.67 (±0.90), indicating that hyoid superior displacement exceeds mylohyoid muscle shortening alone. The median posterior mylohyoid $A{\mathrm{GR}}_{\mathrm{Yp}}$ was 1.22 (±0.20), indicating that hyoid elevation velocity exceeded peak posterior mylohyoid muscle shortening velocity. Notably, in Monkey C, all AGR measurements were significantly greater (*p* < 0.001) when the animal had a craniocervical posture looking left as opposed to looking right during swallowing (means and standard deviations reported in Table 2). Unilateral EMG and kinematic data were obtained from the right mylohyoid. When the animal was looking left, the hyoid was deviated toward the right hemimandible and vice versa.

In contrast, hyoid protraction is not amplified by geniohyoid muscle rotation. The median geniohyoid ${\mathrm{AGR}}_{\underline{X}}$ was 1.00 (±0.14), indicating that hyoid horizontal displacement did not exceed geniohyoid muscle shortening alone. The median geniohyoid $A{\mathrm{GR}}_{\mathrm{Xp}}$ was 1.04 (±0.07), indicating that hyoid protraction velocity did not significantly differ from peak geniohyoid muscle shortening velocity. In Monkey C, craniocervical posture did not significantly affect geniohyoid AGRs (*p* > 0.20).

### 3.3 Hyolingual Muscles Are Primarily Concentrically Active but Also Demonstrate Periods of Isometric and Eccentric Activity During Swallowing

In all four animals, mylohyoid activity began prior to TBR, coincident with the onset of mylohyoid muscle shortening and of hyoid elevation (Figures 2B and 4). Mylohyoid activity peaked before TBR onset and remained activated throughout TBR. Active mylohyoid shortening—concentric activity—continued well into TBR in all animals. In Monkeys C and K, mylohyoid shortening slowed in the latter part of TBR so that activity became essentially isometric (Figure 4).

All four animals showed a burst of anterior digastric muscle activity starting and peaking around minimum gape, synchronous with the start of hyoid elevation and prior to the onset of both jaw depression and TBR (Figure 2C). In the animals with digastric markers, this initial burst of anterior digastric activity was accompanied by marked sagittal rotation (see above) of the anterior digastric as the hyoid elevated such that anterior digastric only shortened prior to TBR in one animal (Monkey J). As hyoid elevation combined with hyoid protraction around the onset of TBR, the anterior digastric continued to rotate in sagittal planes and showed consistent but low‐level EMG activity accompanied by varying degrees of shortening. There was a second burst of anterior digastric activity between the end of TBR and maximum jaw gape associated with further muscle shortening (Figure 4). In the one animal with reliable posterior digastric EMG data (Monkey J), this muscle was concentrically active during hyoid elevation immediately before TBR, minimally active during TBR, and low‐level isometrically active late in the swallow (Figure 4).

Geniohyoid showed similar activity patterns in Monkeys C, J and K: onset in activity prior to TBR followed by a rapid increase in amplitude coincident with both the start of TBR and an increase in hyoid protraction velocity (Figure 2A,D). Geniohyoid activity was similar in Monkey H, but the activity peak prior to TBR looks suspiciously like the mylohyoid activity (cf. Figure 2C), suggesting it may reflect cross‐talk between muscles. Regardless, geniohyoid shortened and hence was concentrically active throughout tongue base retraction in all four animals (Figure 4).

Genioglossus activity and velocity were particularly variable among individual animals (Figure 3B). Three animals showed concentric activity before tongue base retraction (Monkeys C, H, and K), and three showed either a burst or sustained low‐level isometric or eccentric activity during tongue base retraction (Monkeys C, H, and J). The earlier onset of the pre‐retraction genioglossus burst corresponds with the observation that Monkey H formed a trough in the midline tongue earlier in the swallow than the other animals.

Hyoglossus EMG data were not analyzed due to post‐mortem electrode malposition. In the three animals for which kinematic data were successfully collected, hyoglossus shortened as the hyoid elevated and protracted during TBR (Figure 3C). Electrode placement in styloglossus was only verified for one animal: styloglossus shifted from being concentrically active early in the swallow to being primarily eccentrically active during TBR.

Stylohyoid EMG data were also not analyzed due to post‐mortem electrode malposition. In all four animals, stylohyoid rotation during hyoid elevation prior to TBR was accompanied by muscle shortening, and stylohyoid rotation accompanying hyoid protraction during TBR was accompanied by stable or slightly increased stylohyoid length. Therefore, if stylohyoid were active during TBR, it would function iso‐ or eccentrically (Figure 3D) opposite geniohyoid given its orientation.

## 4 Discussion

This study examined the muscular mechanisms underlying hyoid movement during the TBR phase of swallowing in macaque primates by analyzing linear and angular hyolingual muscle kinematics as well as muscle activity mode: that is, concentric, eccentric, or isometric activity. Although inquiries into the functional consequences of static and dynamic muscle geometry have advanced the field of muscle functional morphology of other musculoskeletal systems (Gans and Bock 1965; Azizi et al. 2008), this is the first time to our knowledge that whole‐muscle rotational kinematics have been studied in the hyolingual apparatus or other musculoskeletal apparati without the kinematic constraints of joint surfaces (e.g., thoraco‐abdominal or pelvic diaphragms).

### 4.1 A New Perspective on the Kinematic Chain: A Proposed Pulley Paradigm

Our results are consistent with the hypothesis that hyoid elevation and protraction, which cause the macaque tongue base to retract (Orsbon et al. 2020), are driven by both active shortening and rotation of the mylohyoid, active rotation of the digastric muscle‐tendon unit, and active shortening of the geniohyoid. To understand how the observed kinematics produce hyoid movement, we characterize the suprahyoid muscles and hyoid as a simple but multiple pulley apparatus tethered at a shared point—the hyoid. The system could also be modeled as a load suspended by multiple linear actuators with mobile insertion points. In this system, the instantaneous position of the hyoid at the center of a stellate muscular suspensorium depends on the summation of loads imparted on the hyoid by active and passive muscle tension. The apparatus is morphologically straightforward, yet it is nonetheless biomechanically complex due to the interconnected nature of the craniofacial kinematic chain (Hiiemae and Palmer 2003). For example, the geniohyoid and posterior mylohyoid muscles have fixed anchors on the mandible, yet the mandible itself moves relative to the cranium and the hyoid. Overall, the power of the pulley analogy is its ability to highlight that shortening of a single muscle will result in not only hyoid motion but also reorientation of all other muscles attached to the hyoid.

Within this framework, muscle activity is proposed to generate hyoid motion during swallowing as follows: Bilateral posterior mylohyoid muscles actively shorten and rotate to both synergistically elevate the hyoid and antagonistically neutralize each other's laterally oriented forces. A key exception may be while the head is turned, as noted in Monkey C. Given that the hyoid would deviate back toward the midline when looking in either direction, forces produced by these muscles may be modulated passively through architectural gearing mechanics due to differential loads on the mylohyoid by infrahyoid forces that resist rotation of the hyoid laterally with the mandible as the head turns.

Simultaneous with posterior mylohyoid activity, variable patterns of anterior and posterior digastric muscle activity between animals resulted in linearization of the overall digastric muscle‐tendon unit among all animals, which may facilitate hyoid elevation analogous to the effect of a movable pulley when applied forces result in upward displacement of the pulley. After hyoid elevation onset, sustained but decreased posterior mylohyoid and digastric muscle activity continues hyoid elevation as geniohyoid ramps up activity.

The co‐linear bilateral geniohyoid muscles produce nearly identical movements and so function synergistically to protract the hyoid closer to the mandibular symphysis. Geniohyoid does rotate, but this appears passive and due to bilateral posterior mylohyoid shortening and rotation and possibly linearization of the digastric rather than through gearing intrinsic to the muscle. In contrast, right and left mylohyoid as well as the anterior and posterior digastric bellies are their own synergists and antagonists to drive hyoid elevation. Geniohyoid active shortening produces some passive rotation on posterior mylohyoid in the sagittal plane, given that the posterior mylohyoid rotates anterior to its insertion point by the end of TBR. An additional possibility is active rotation by the anterior mylohyoid fibers transmitted to the hyoid via the midline raphe. However, these muscles were not assessed in this experiment due to methodological constraints.

Although stylohyoid EMG data could not be used due to electrode migration, observed shortening corresponding with mylohyoid activity could be active or passive depending on the amount of tension within the muscle before hyoid elevation onset. Although the stylohyoid and geniohyoid slightly linearize analogous to the digastric muscles, the geniohyoid is a much larger muscle than the stylohyoid and would likely overpower any antagonistic forces generated by the smaller stylohyoid.

Extrinsic lingual muscles also rotate during swallowing. Genioglossus rotation mirrors that of geniohyoid and is favored to be a passive product of mouth floor elevation rather than active, especially given that most animals' genioglossus muscle was eccentrically active during TBR. Hyoglossus may function as an additional fixed pulley on the hyoid in swallowing given its minimal change in orientation or lingual insertion position as it shortens during TBR. However, outside of swallowing, the hyoglossus may be afforded additional mobility by its lingual insertion, but such a hypothesis will require additional study incorporating EMG in behaviors that involve more movement of the lateral tongue, for example, chewing.

As noted in prior work, greater gearing and fiber rotation comes at the cost of muscle force production (Azizi et al. 2008). The negation of forces by contralateral antagonism that produces rotation in the bilateral posterior mylohyoid bellies as well as the unilateral antagonism of the anterior and posterior digastric bellies suggests that these muscles function to prioritize displacement and/or velocity over force, especially given the lack of resistance to initial hyoid elevation by the bolus within the oral cavity. Future work would be required to assess potential antagonistic forces generated by the infrahyoid muscles, especially given that variable gearing within the posterior mylohyoid in Monkey C suggests that its gearing is load‐controlled in response to tension from infrahyoid muscle antagonists, as has been observed by others (Azizi et al. 2008). In contrast, the minimal rotation in geniohyoid and hyoglossus with no significant antagonists to these muscles suggests that these muscles function to prioritize both displacement and force, that is, power, to overcome resistance imposed by the bolus friction and inertia as well as lingual tissue inertia to force the tongue base and bolus posteriorly. In summary, our data support the hydraulic hypothesis of hyoid kinematics driving TBR, which is powered by: active shortening and rotation of mylohyoid and linearization of the digastric that optimizes muscle velocity to quickly “prime the pump” in a low resistance setting, and subsequent active shortening of geniohyoid (and possibly hyoglossus) that optimizes muscle power to drive the hydraulic mechanism in a higher resistance setting.

### 4.2 Muscle Rotation Can Amplify or Reduce Hyoid Kinematics Depending on Geometry

Hyolingual muscle geometry and the direction of rotation combine to determine whether rotation significantly amplifies or depresses movement. As demonstrated in Table 2, rotation amplifies hyoid elevation beyond that of muscle shortening alone. In contrast, rotation has no significant effect or even decreases hyoid protraction relative to muscle shortening. Other studies have demonstrated architectural gear ratios greater than or equal to 1.00 (Brainerd and Azizi 2005; Azizi and Brainerd 2007; Azizi et al. 2008; Azizi and Roberts 2014; Holt et al. 2016). To our knowledge, ours is the first study to demonstrate that an AGR can be less than 1.00 in vivo, as it was in Monkeys H and J, implying that geniohyoid muscle rotation can reduce hyoid protraction relative to muscle shortening. However, the functional significance of this is likely to be negligible given the ${\mathrm{AGR}}_{\underline{X}}$ of 1.00 (0.14) and $A{\mathrm{GR}}_{\mathrm{Xp}}$ of 1.04 (0.07).

Whether muscle rotation amplifies hyoid kinematics depends on whether rotation occurs away or toward from the axis of movement. Mylohyoid amplifies hyoid elevation as its orientation rotates away from the superoinferior (Y) axis and toward the mediolateral (Z) axis in the coronal plane. Moreover, mylohyoid muscle at rest has an intermediate angle relative to these axes, and its higher gear ratio is consistent with a similar study by Brainerd and Azizi (2005) which modeled the effects of initial angle on architectural gear ratios. For rotating muscle fibers, the less a muscle is aligned with the axis of movement, the more rotations amplify displacement along that axis at the expense of force generated along that axis.

Just as rotation away from an axis can amplify movement, rotation toward an axis can reduce movement. In Monkeys H and J, the geniohyoid hyoid attachment was nearly always inferior to the geniohyoid mandibular attachment. Consequently, geniohyoid shortened more than the hyoid protracted because elevation rotated geniohyoid toward the anteroposterior (X) axis. In contrast, the geniohyoid hyoid attachment was higher in Monkeys C and K, and these animals had more hyoid protraction than geniohyoid shortening because elevation rotated geniohyoid away from the anteroposterior axis.

In summary, these findings emphasize the importance of a dynamic perspective on suprahyoid muscle biomechanics. As muscles actively shorten, they can produce muscle rotation through antagonism with other suprahyoid muscles while simultaneously producing hyoid translation synergistically. As the hyoid translates, all suprahyoid muscle orientations change, and the functional significance of these changes depends on the original orientation of the muscles, that is, hyoid posture (German et al. 2011, Li et al. 2025). Within such a dynamic and geometric approach to suprahyoid biomechanics, hypotheses regarding the consequences of craniofacial morphology and hyoid posture variation on swallowing biomechanics over the course of human evolution can be quantitatively tested.

### 4.3 Limitations

This study has several limitations. The lengthy processing time for bi‐planar videoradiographic data limited data analysis to four animals and one food type (red grapes). Although infrahyoid muscles are complex and are likely to be an important determinant of hyoid excursion (Konow et al. 2010; Wentzel et al. 2011; Yamazaki et al. 2017), this study did not evaluate infrahyoid muscle function. The manubrium sternum—where sternohyoid and sternothyroid insert—often was not included in the field of view or was not clearly visible, thus rendering infrahyoid muscle length measurements unreliable given our experimental design. Revision of the primate chair design, or data collection in unrestrained animals, would allow the functional morphology of these important antagonistic muscles to be examined in future work.

## 5 Conclusion

This high‐resolution interrogation of macaque hyolingual muscle function demonstrates that the hypothesized hydraulic mechanism of tongue base retraction (TBR) during swallowing is generated not only by active suprahyoid muscle shortening but also posterior mylohyoid and digastric belly rotation. Prior to TBR, posterior mylohyoid muscle active shortening and rotation and possibly hyoglossus shortening compress the tongue and suprahyoid tissues against the rigid structures of the oral cavity—essentially, “priming the pump.” This compression is further facilitated by rotation of the digastric muscles and active anterior digastric shortening, resulting in digastric linearization that functions analogous to a movable or compound pulley in engineering. The amplified hyoid kinematics associated with posterior mylohyoid rotation suggest that this muscle's function prioritizes displacement and velocity in a system with low resistance to hyoid elevation. Once the tongue and hyoid are “primed” by the posterior mylohyoid and digastric muscles, geniohyoid and possibly further hyoglossus muscle shortening power hydraulic TBR primarily through active shortening, suggesting functional prioritization of force to convert hyoid protraction into TBR and bolus propulsion via a hydraulic linkage. Because of the lack of joint constraints and geometry of suprahyoid muscle attachment points, all suprahyoid muscles rotate to some extent as they and/or other muscles shorten. However, whether muscle rotation amplifies or even reduces movements along a given axis depends on whether a muscle rotates away from or toward a given axis of movement, respectively, indicating that functional assessment of suprahyoid muscle rotation necessitates correlation with hyoid kinematics (German et al. 2011). In conclusion, variation in hyolingual and craniofacial morphology and their consequent effects on muscle geometry in this floating system may have an underappreciated role in the comparative biomechanics and functional morphology of swallowing, hinting at possible morphological integration that may have shaped the human face and hyolingual apparatus.

## Author Contributions

**Courtney P. Orsbon:** conceptualization, investigation, funding acquisition, writing – original draft, methodology, validation, visualization, writing – review and editing, software, formal analysis, data curation. **Nicholas J. Gidmark:** supervision, methodology, conceptualization, investigation, writing – review and editing. **Callum F. Ross:** conceptualization, investigation, methodology, writing – review and editing, supervision, resources, formal analysis, project administration, funding acquisition.

## Funding

This study was supported by the NIH (NIDCR and NICHD); NSF (MRI and BCS‐DDRIG). Grant numbers: R01‐DE023816 and T32‐HD009007 (NIH); DBI‐1338066 and BCS‐1732175 (NSF).

## Conflicts of Interest

The authors declare no conflicts of interest.

## Data Availability

The data that support the findings of this study are available from the corresponding author upon reasonable request.
